# 

**Published:** 1893-02

**Authors:** 


### CONTENTS.

COMMUNICATIONS.

Empyema of the Maxillary Sinus.......... 53
Post-mortem Treatment of the Dental Pulp.......... 65
Clinical Lecture upon Hare-Lip, Cleft Palate and Epithelioma.......... 73
The Saliva, from a Dental Standpoint.......... 79
Diseases of the Gums.......... 81

SELECTIONS.

Medical Charlatans.......... 72
Eyesight: Its Care During Infancy and Youth.......... 88
The Teaching Element in Congresses.......... 95
Two New Local Anæsthetics.......... 96

THE COLUMBIAN DENTAL CONGRESS.

The Columbian Dental Congress.......... 97
The Dental Congress.......... 98
International Medical Congress.......... 99
Pan-American Medical Congress.......... 100

EDITORIAL.

Bibliography.......... 101
Postponement.......... 103
Bibliographical.......... 103
Biographical.......... 104

